# Effects of SARS-CoV-2 infection on health and functional capacity in institutionalized older adults

**DOI:** 10.1590/1980-220X-REEUSP-2023-0128en

**Published:** 2023-12-22

**Authors:** Bárbara Lima Queiroz, Carlos Queiroz do Nascimento, Thamires Otaviano Marques de Souza, Gabriel Soares Bádue, Nassib Bezerra Bueno, Sandra Mary Lima Vasconcelos, Carolina Santos Mello, Müller Ribeiro-Andrade, Terezinha da Rocha Ataíde, João Araújo Barros-Neto

**Affiliations:** 1 Faculdade Estácio de Alagoas, Departamento de Enfermagem, Maceió, AL, Brazil.; 2 Universidade Federal de Alagoas, Faculdade de Nutrição, Maceió, AL, Brazil.; 3 Universidade Federal de Alagoas, Instituto de Ciências Biológicas e da Saúde, Maceió, AL, Brazil.

**Keywords:** Aged, COVID-19, Functional Status, Nutritional Status, Anciano, COVID-19, Estado Funcional, Estado Nutricional, Idoso, COVID-19, Estado Funcional, Estado Nutricional

## Abstract

**Objective:**

To assess the effect of SARS-CoV-2 infection on the health conditions and functional capacity of older adults living in long-term care units in Maceió City – Alagoas State.

**Methods:**

A prospective cohort was conducted with institutionalized older adults of both sexes. Older adults were assessed for clinical conditions (diagnosis of chronic diseases and biochemical tests), functional capacity, and nutritional status. All assessments were repeated on two occasions, maintaining a 6-month interval between them.

**Results:**

The sample was composed of 289 older adults. Of the total, 98 (33.9%) were positive for COVID-19 and eight died (2.8%). Men were more likely to have COVID-19 (OR = 3.50; p < 0.01). It was observed that the disease contributed to increasing the frequency of dependent older adults after six months (OR = 1.38; p-interaction < 0.01). It was also observed that after six months of positive diagnosis for COVID-19, there was greater weight loss (p < 0.01), reduced BMI (p < 0.01), increased mean SBP (p = 0.04), and DBP (p = 0.03).

**Conclusion:**

Effects of COVID-19 in institutionalized older adults go beyond acute complications and compromise blood pressure control, functional capacity, and favor weight loss.

## RESUMO

**Objetivo:**

Avaliar o efeito da infecção pelo SARS-CoV-2 nas condições de saúde e capacidade funcional de idosos residentes em unidades de longa permanência na cidade de Maceió – Alagoas.

**Métodos:**

Foi realizada uma coorte prospectiva com idosos institucionalizados de ambos os sexos. Os idosos foram avaliados quanto às condições clínicas (diagnóstico de doenças crônicas e exames bioquímicos), capacidade funcional e estado nutricional. Todas as avaliações foram repetidas em duas ocasiões, mantendo um intervalo de 6 meses entre elas.

**Resultados:**

A amostra foi composta por 289 idosos. Do total, 98 (33,9%) deram positivo para COVID-19 e oito faleceram (2,8%). Os homens tiveram maior probabilidade de ter COVID-19 (OR = 3,50; p < 0,01). Observou-se que a doença contribuiu para o aumento da frequência de idosos dependentes após seis meses (OR = 1,38; interação de p <0,01). Observou-se também que após seis meses de diagnóstico positivo para COVID-19, houve maior perda de peso (p < 0,01), redução do IMC (p < 0,01), aumento da média da PAS (p = 0,04) e da PAD (p = 0,03).

**Conclusão:**

Os efeitos da COVID-19 em idosos institucionalizados vão além das complicações agudas e comprometem o controle da pressão arterial, a capacidade funcional, favorecendo a perda de peso.

## RESUMEN

**Objetivo:**

Evaluar el efecto de la infección por SARS-CoV-2 sobre las condiciones de salud y la capacidad funcional de personas mayores que viven en unidades de cuidados a largo plazo en la ciudad de Maceió – Alagoas.

**Métodos:**

Se realizó una cohorte prospectiva con ancianos institucionalizados de ambos sexos. Los ancianos fueron evaluados en cuanto a condiciones clínicas (diagnóstico de enfermedades crónicas y pruebas bioquímicas), capacidad funcional y estado nutricional. Todas las evaluaciones se repitieron en dos ocasiones, manteniendo un intervalo de 6 meses entre ellas.

**Resultados:**

La muestra estuvo compuesta por 289 personas mayores. Del total, 98 (33,9%) dieron positivo a la COVID-19 y ocho fallecieron (2,8%). Los hombres tenían más probabilidades de tener COVID-19 (OR = 3,50; p <0,01). Se observó que la enfermedad contribuyó al aumento de la frecuencia de ancianos dependientes después de seis meses (OR = 1,38; interacción p < 0,01). Se observó que la enfermedad contribuyó al aumento de la frecuencia de ancianos dependientes después de seis meses (OR = 1,38; interacción p < 0,01). También se observó que después de seis meses de diagnóstico positivo para COVID-19, hubo mayor pérdida de peso (p < 0,01), reducción del IMC (p < 0,01), aumento de la PAS media (p = 0,04) y la PAD (p = 0,03).

**Conclusión:**

Los efectos del COVID-19 en ancianos institucionalizados van más allá de las complicaciones agudas y comprometen el control de la presión arterial, la capacidad funcional, favoreciendo la pérdida de peso.

## INTRODUCTION

In December 2019, the virus designated Severe Acute Respiratory Syndrome Coronavirus-2 (SARS-CoV-2) was first identified in the Chinese city of Wuhan. The first cases probably appeared in a seafood market, but evidence is still fragile about the location and source of the infections^(1)^.

At the beginning of the spread of the virus in several countries, the World Health Organization (WHO) announced on January 30, 2020 that the coronavirus disease (COVID-19)constituted a Public Health Emergency of International Concern (PHEIC). On March 11, 2020, the installed health situation was classified as a pandemic. By then, there was a scenario of deaths and thousands of confirmed cases of COVID-19 on all continents, and older adults were the most affected^(2,3)^.

Older adults are considered to be the population group most at risk of clinical complications of COVID-19, especially among those living in Nursing Homes (NHs), as they are subject to multimorbidity, since in these institutions there is a higher frequency of frail older adults with greater functional dependence, culminating in greater difficulty in recovering health after infectious processes, such as infection by SARS-CoV-2^(4)^.Internationally, at the beginning of the first year of the pandemic, high mortality rates from the disease were recorded in NHs in several countries. Among the older adults who tested positive for COVID-19 in these institutions, about 19% died in Australia, while Portugal and France had the highest mortality rates of 40% and 51%, respectively^(5)^. In Brazil, this data is not yet available. The course of COVID-19 infection in older adults is heterogeneous. It shows significant variations, both in the acute phase of the disease and in the recovery phase, where some people may progressively develop functional limitations and dependence after infection^(6)^. This condition, in turn, can result in a situation of functional incapacity and/or dependence that manifests itself in difficulty performing basic activities of daily living (BADL), potentially leading to the development of geriatric syndromes such as postural instability, immobility, incontinence and communicative incapacity, conditions that compromise quality of life, reducing life expectancy and keeping older adults more vulnerable to infections and other pathological processes^(7)^.

Although the literature has already shown an association between functional status prior to SARS-CoV-2 infection and adverse outcomes of the disease in older adults^(8)^, there is still a lack of clinical data on the care needs and the impact of functional capacity of older people after the acute phase of COVID-19.

Studies involving older adults living in NHs are still incipient and cannot reflect the consequences of COVID-19 on the health of survivors. Thus, identifying the impact of COVID-19 on functional capacity and the need for post-infection care is of crucial importance for establishing perspectives and prognoses for institutionalized older adults’ health, assisting in nursing care planning and health resource management as well as allowing for a more accurate prediction of the need for rehabilitation. In this context, this study aimed to assess the effect of SARS-CoV-2 infection on the health conditions and functional capacity of older adults living in NHs in Maceió City – Alagoas State.

## METHOD

### Design and Setting

This is a prospective cohort study conducted with older adults living in NHs in Maceió City, using the STrengthening the Reporting of OBservational studies in Epidemiology (STROBE) tool as a guide.

### Local, Sample and Selection Criteria

The sampling plan of this study consisted of a non-probability convenience sampling, where initially, the universe of older adults residents in NHs of Maceió registered in the home care service (HCS) of the Municipal Health Department of Maceió (MHD).

The patients included in the study were identified from the visit to the institutions. Older adults living in any of the Maceió NHs, aged 65 years or older, who agreed to be tested for COVID-19 at the time of the first visit and have not previously been diagnosed with COVID-19 were included. Older adults with a confirmed diagnosis of COVID-19 before or after the first assessment, not having participated in the second assessment and having incomplete clinical, sociodemographic and/or anthropometric data were excluded.

### Data Collection

The study was conducted from April 2020 to November 2020, and data was collected in two stages. The first phase of data collection occurred between April and May 2020. The second assessment was performed between October and November 2020, respecting the minimum periodicity of 6 months after the first assessment in NHs.

All the older adults were tested for COVID-19 during their first visit (baseline), and sociodemographic variables, lifestyle, and health conditions (current and past) were collected. Blood samples were also taken for clinical assessment, and anthropometric and functional capacity assessments were carried out.

After testing for COVID-19, participants were divided into two groups: one made up of all older adults who tested positive for COVID-19 (COV-g) and the other made up of people who tested negative for the disease (NCOV-g).

In the second (deadline) assessment, carried out 6 months after the first, same variables were collected as in the first visit, following the same instruments and assessment protocols, and all older adults were retested for COVID-19.

### Sociodemographic and Lifestyle Data

Sociodemographic data were collected at the first visit to NHs when older adults were identified. In this study, the following sociodemographic variables were collected: age (categorized as <80 years and ≥80 years); gender; education (categorized as <4 years and >4 years of study); and marital status (categorized as “with companion or married” and “single, divorced, or widowed”).

Lifestyle assessment was performed by assessing the habit of alcohol consumption, smoking, and report of physical activity, and all those who reported using alcoholic beverages, even if rarely (<1 time/month), were considered drinkers as well as those who reported never drinking or those who reported having stopped using alcoholic beverages at least 30 days ago were considered nonsmokers. Regarding smoking, we classified smokers as those who reported smoking, regardless of frequency and nonsmokers those who quit smoking at least 30 days ago or never smoked at all. The older adults who engaged in physical activity at NHs were considered physically active, regardless of intensity.

### Clinical Assessment

The history of pre-existing chronic diseases such as hypertension, diabetes, chronic obstructive pulmonary disease (COPD), cardiovascular disease, dementia syndrome, and other diseases with previously established medical diagnoses was investigated.

Blood pressure (BP) measurement was performed in duplicate during the two stages of the study, with individuals seated and after five minutes of rest using Omron HEM-742^®^ digital devices. This assessment followed the protocol and reference standard of the updated Brazilian Society of Cardiology Guidelines for Cardiovascular Prevention^(9)^.

Biological material (blood) was collected by puncture from the basilica or cubital vein through a vacuum system, using tubes with a capacity of 6 ml, and the material was placed in an EDTA K^3^ (5mL) of BD^®^ brand, correctly identified for biochemical tests in the two assessments.

Hematological analyses for hemoglobin, hematocrit, leukocytes, and lymphocytes were processed in the Cell-Dyn Ruby hematological analyzer, commercialized by ABBOTT^®^, duly calibrated, and following the manufacturer’s norms and recommendations^(10)^.

Blood glucose was measured with older adults fasting by the colorimetric enzymatic glucose (GE-C) laboratory method with venous blood using the glucose reagent kit from the manufacturer Labtest *Diagnóstica* ^®^.

The ultraviolet (UV) method reaction performed the serum urea dosage. The reference values considered normal were 10 to 50 mg/dl.

Serum creatinine assessment was performed by the kinetic method reaction and calibrated according to the laboratory method. The reference values considered were 0.40 to 1.40 mg/dl.

### Covid-19 Diagnosis

Diagnosis for COVID-19 was performed at the two on-site visits by means of the “rapid” immunological test from the SARS-CoV-2 Antigen Test^(11)^.

### Anthropometric Assessment

Similarly, anthropometric assessments were carried out at the time of admission to the study and after six months.

Weight was estimated from the equation validated for older adults residents in NHs by Jung et al.^(12)^.

Stature was estimated from the knee height (KH) measurement and applied in a formula, according to Chumlea et al.^(13)^.

KH was measured with the help of an anthropometric ruler with a metal rod. Older adults kept the leg bent, forming a 90° angle with the knee. The fixed part of the ruler was placed under the heel, and the mobile part was brought to the suprapatellar region.

Arm circumference was measured at the midpoint of the non-dominant arm using an inextensible, flexible tape measure, and the results were assessed according to the Third National Health and Nutrition Examination Survey (NHANES III) reference values (1988-1994)^(14)^.

Calf circumference was assessed at the greatest circumference between the ankle and the knee, following the protocol and cut-off point established by the WHO; values <31cm indicated a loss of muscle mass^(15)^.

The Lipschitz criteria was adopted to classify older adults’ nutritional status based on Body Mass Index (BMI) values^(16)^.

Unintentional weight loss was also assessed, identified by the difference between the weight assessed at the first and second visit, being considered a frailty indicator weight loss greater than 4.5 kg or a loss of more than 5% of the initial weight^(17)^.

### Functional Assessment

The assessment of older adults’ functional capacity was performed with no baseline and no deadline using the Barthel index in association with the questionnaire that assesses BADL, as Lawton and Brody recommended^(18)^ and classified as dependent or independent.

### Data Analysis and Treatment

The behavior of the variables was checked for normality distribution (Kolmogorov-Smirnov test with Lilliefors correction) and for homogeneity of variance of residuals (Levene test).

To verify the association between the frequency of categorical variables among the older adults with and without COVID-19 diagnosis, Pearson’s chi-square or Fisher’s exact test was performed.

To investigate whether COVID diagnosis influenced the variation in older adults’ functional capacity diagnosis over time, univariable logistic regressions were conducted at each time point to calculate the Odds Ratio of the group with COVID presenting the “dependent” classification compared to the group without COVID, in the first and second assessment. Finally, to verify whether the variation in the “dependent” classification differed significantly between the group with COVID compared to the group without COVID, the interaction between the Odds Ratios at the first and second time points was calculated using a generalized estimating equation (GEE, via the GENLIN command), adjusted for sex, age, education, drinking, smoking, physical activity, diagnosis of chronic diseases (hypertension, diabetes, COPD, and cardiovascular disease).

To verify the influence of COVID-19 diagnosis on the other health markers of this study as a function of time, we initially identified the difference between means of the variables in each group [(final - initial = delta (∆)]. Next, an uncontrolled analysis was performed using t-test for comparison of means for independent variables to verify whether the deltas differed between the groups of older adult individuals with and without the disease. Finally, an analysis of covariance (ANCOVA) was conducted, adjusted for sex, age, education, alcoholism, smoking, physical activity, diagnosis of chronic diseases (hypertension, diabetes, COPD, and cardiovascular disease).

All analyses were conducted using SPSS v24.0 (IBM Inc, Chicago, IL) and an alpha value of 5% was adopted. Cramer’s V for Pearson’s chi-square test, Cohen’s d for t-test and ω^2^ for ANCOVA were used to assess the effect size.

### Ethical Aspects

The study was developed following the recommendations of Resolution 466/2012 of the Brazilian National Health Council, and was approved by the Research Ethics Committee (REC) of the *Universidade Federal de Alagoas*, via Brazil platform, under Opinion 4.314.080/2020. Eligible patients and study participants signed, beforehand, the Informed Consent Form.

## RESULTS

### Characterization and Sample

**Figure 1:**
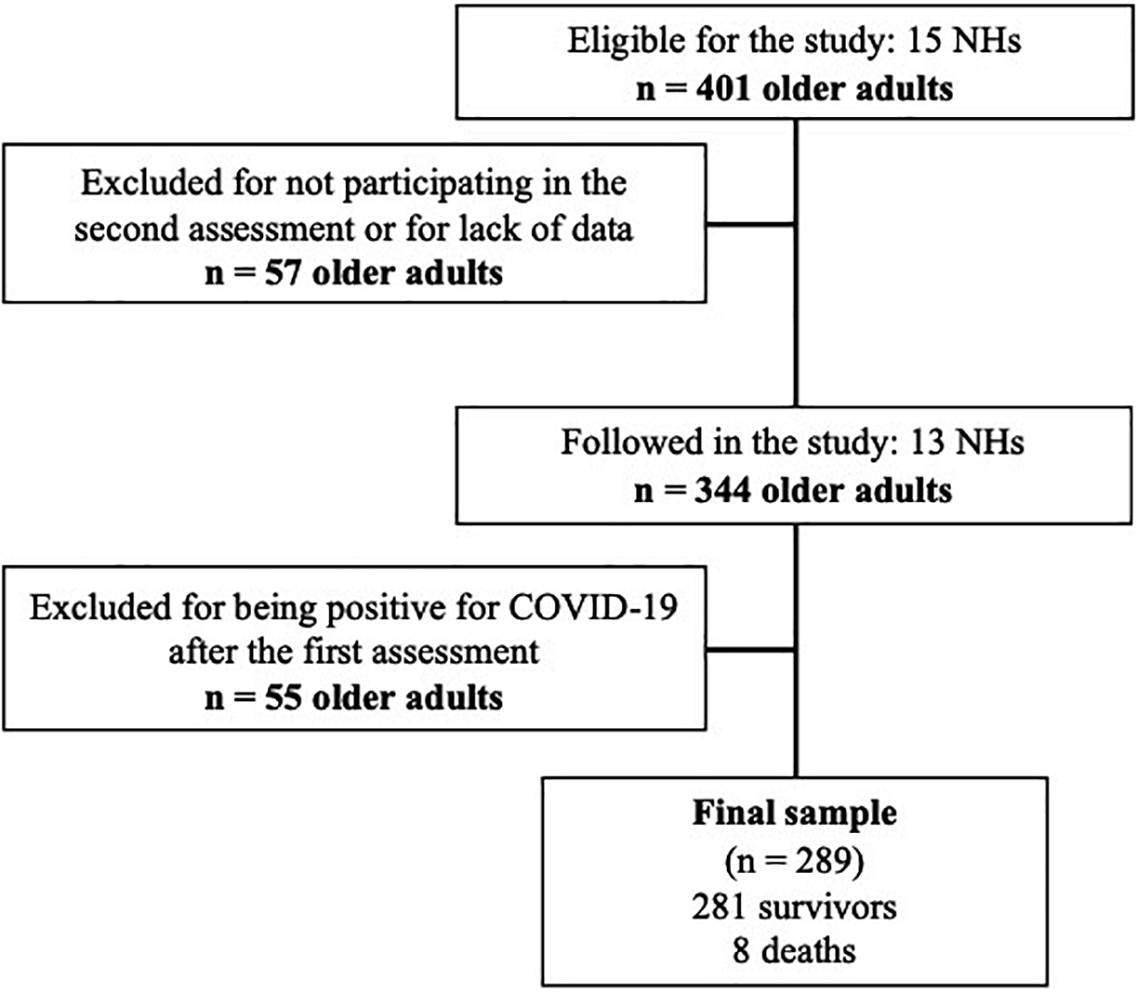
Flowchart of sample definition after exclusion criteria.

Altogether, 289 older adults comprised the final sample (Figure 1), of whom 98 (33.9%) were positive for COVID-19 in the first assessment and 8 (2.8%) died. Most of the sample was composed of women (n = 164; 56.7%), and almost half of the sample was 80 years of age or older (n = 140; 48.4%) (Table 1).

**Table 1:** Socio-demographic and clinical characterization of older adults living in NHs (n = 289) – Maceió, AL, Brazil, 2021.

Variables	NCOV-g (n = 191)		COV-g (n = 98)		OR	p-value	Cramer’s V
	N	%	N	%			
Age							
<80 years	102	53.4	47	48.0	1.25	0.38*	0.05
≥80 years	89	46.6	51	52.0			
Sex (female)	128	67.0	36	36.7	3.50	<0.01*	0.29
Marital status (without partner)	175	91.6	90	91.8	0.97	0.95*	0.01
Education (≤4 years)	125	65.4	51	52.0	0.57	0.03*	0.13
Alcohol consuption (yes)	8	4.2	7	7.1	1.76	0.28*	0.06
Smoker (yes)	30	15.7	17	17.3	1.13	0.72*	0.02
Physical activity (inactive)	144	75.4	68	69.4	0.74	0.27*	0.06
Diag. of hypertension (yes)	92	48.2	56	57.1	1.44	0.15*	0.09
Diag. of diabetes (yes)	43	22.5	29	29.6	1.45	0.19*	0.08
Diag. of CVD (yes)	12	6.3	8	8.2	1.33	0.55*	0.04
Diag. of COPD (yes)	7	3.7	4	4.1	1.12	0.55^#^	0.01
Diag. of demencia (yes)	17	8.9	10	10.2	1.16	0.72*	0.02
Diag. of other CD (yes)	46	24.1	20	20.4	0.81	0.48*	0.04

NCOV-g: negative for COVID-19; COV-g: positive for COVID-19. OR: unadjusted Odds Ratio; COVID: coronavirus disease; CVD: cardiovascular disease; COPD: chronic obstructive pulmonary disease; CD: chronic disease; Diag: diagnosis.

* p-value obtained by Pearson’s chi-square test; #p-value obtained by Fisher’s exact test.

It was observed that among older adults in the group with positive result for the disease, the majority was male (OR = 3.50; p < 0.01; Cramer’s V = 0.29) and had less education (p = 0.03; Cramer’s V = 0.13).

### Effects of Covid-19 on Functional Capacity in Institutionalized Older Adults After Six Months of Infection

It was observed that the COV-g group at the beginning of the study had a lower frequency of dependent older adults when compared to the NCOV-g group (53.3% vs. 57.6%; OR = 0.84), and after six months of diagnosis this frequency was reversed in the COV-g (66.7% vs. 59.2%; OR = 1.38).

When testing the interaction of functional dependence as a function of COVID-19 over time by univariable analysis, it was identified that the disease contributed significantly to the increase in the “dependent” classification among older adults in COV-g when compared to NCOV-g (p-interaction < 0.01) (Table 2).

This result was maintained in the multivariable analysis, showing that the increase in functional dependence after six months of COVID-19 diagnosis was significantly higher than in the group without the diagnosis (p-interaction < 0.01) (Table 2).

**Table 2:** Analysis of association between COVID-19 diagnosis and functional capacity in older adults before and after six months of diagnosis (n = 281) – Maceió, AL, Brazil, 2021.

Functional capacity		NCOV-g (n = 191)		COV-g (n = 90)		OR	95%CI	p-value*	p-interaction^#^
		N	%	N	%				
**Univariable analysis** ^a^									<0.01
Initial	Independent	81	42.4	42	46.7	1	–	–	
	Dependent	110	57.6	48	53.3	0.84	0.5; 1.39	0.50	
Final	Independent	78	40.8	30	33.3	1	–	–	
	Dependent	113	59.2	60	66.7	1.38	0.81; 2.33	0.22	

**Multivariable analysis** ^b^									<0.01
Initial	Independent	81	42.4	42	46.7	1	–	–	
	Dependent	110	57.6	48	53.3	0.67	0.37; 1.22	0.19	
Final	Independent	78	40.8	30	33.3	1	–	–	
	Dependent	113	59.2	60	66.7	1.27	0.69; 2.34	0.44	

NCOV-g: negative for COVID-19; COV-g: positive for COVID-19.

^a^OR: Odds Ratio referring to the group with COVID presenting the diagnosis of functional capacity dependence compared to the group without COVID. *p-value obtained through univariable logistic regression. ^#^p-value for the interaction between the Odds Ratio obtained at the initial and final time points, calculated using generalized estimating equations.

^b^OR: Odds Ratio adjusted for sex, age, education, smoking, and diagnoses of hypertension, DM, CVD and COPD, referring to the group with COVID presenting functional capacity classification as “dependent” in relation to the group without COVID. *p-value obtained by multivariable logistic regression. ^#^p-value for the interaction between the adjusted Odds Ratio obtained at the initial and final time points, calculated using generalized estimating equations.

95%CI: 95% confidence interval.

### Effects of Covid-19 on Institutionalized Older Adults’ Nutritional Status and Health Conditions After Six Months of Infection

Comparing the deltas of the variables between groups, higher mean unintentional weight loss (UIWL) was observed in COV-g compared to NCOV-g (–1.02 + 1.79 kg vs. –0.38 + 1.27 kg, respectively; p < 0.01; Cohen’s d = 0.35). Similarly, the reduction in BMI was greater in COV-g (–0.42 + 0.74 kg/m^2^ vs. –0.15 + 0.52 kg/m^2^; p < 0.01; Cohen’s d = 0.35) (Table 3).

**Table 3:** Association analysis between COVID-19 diagnosis with anthropometric measures and clinical variables in institutionalized older adults before and after six months of diagnosis (n = 281) – Maceió, AL, Brazil, 2021.

	NCOV-g (n = 191)		COV-g (n = 90)		p-value	Effect size
	Mean	SD	Mean	SD		
**Crude analysis** ^a^						
ΔWeight (kg)	–0.38	1.27	–1.02	1.79	<0.01	0.35*
ΔBMI (kg/m^2^)	–0.15	0.519	–0.42	0.74	<0.01	0.35*
ΔWC (cm)	–0.19	1.17	–0.21	0.57	0.87	0.01*
ΔSBP (mmHg)	–0.13	5.28	1.33	4.75	0.02	0.94*
ΔDBP (mmHg)	–0.17	6.17	1.67	8.43	0.07	0.35*
ΔHemoglobin (g/dL)	–0.31	1.97	–0.51	2.09	0.45	0.18*
ΔHematocrit (%)	–0.76	4.98	–0.79	5.41	0.97	0.04*
ΔLeukocytes (cont/uL)	–19.96	2679.74	38.74	1268.55	0.87	0.08*
ΔLymphocytes (cont/mm^3^)	–90.31	854.60	–85.33	1268.55	0.97	0.01*
ΔGlucose (mg/dL)	–1.06	66.01	–1.47	69.66	0.96	0.02*
ΔBlood urea (md/dL)	–1.44	17.99	2.86	16.97	0.06	0.23*
ΔCreatinine (md/dL)	0.15	1.68	0.07	0.33	0.64	0.06*

**Adjusted analysis** ^b^						
ΔWeight (kg)	–0.38	1.27	–1.02	1.79	<0.01	0.02**
ΔBMI (kg/m^2^)	–0.15	0.52	–0.42	0.74	<0.01	0.02**
ΔWC (cm)	–0.19	1.17	–0.21	0.57	0.65	0.01**
ΔSBP (mmHg)	–0.13	5.28	1.33	4.75	0.04	0.16**
ΔDBP (mmHg)	–0.17	6.17	1.67	8.43	0.03	0.02**
ΔHemoglobin (g/dL)	–0.31	1.97	–0.51	2.09	0.56	0.01**
ΔHematocrit (%)	0.76	4.98	0.79	5.41	0.98	0.01**
ΔLeukocytes (cont/uL)	–19.96	2679.74	38.72	2817.81	0.52	0.01**
ΔLymphocytes (cont/mm^3^)	–90.31	854.60	–85.33	1268.55	0.48	0.01**
ΔGlucose (mg/dL)	–1.06	66.01	–1.74	69.66	0.78	0.01**
ΔBlood urea (md/dL)	–1.44	17.99	2.86	16.97	0.07	0.01**
ΔCreatinine (md/dL)	0.15	1.68	0.07	0.33	0.56	0.01**

NCOV-g: negative for COVID-19; COV-g: positive for COVID-19. SD = standard deviation; 95% CI: 95% confidence interval; *Cohen’s d; **ω^2^; ^a^p-value obtained using t-test for independent variables. ^b^p-value obtained by analysis of covariance (ANCOVA) adjusted for sex, age, education, smoking and diagnoses of hypertension, DM, CVD and COPD.

The median percent UIWL after six months of COVID-19 diagnosis was higher in the group that tested positive for the disease (3.00%; IQ = 3.50 vs. 2.37%; IQ = 2.3, respectively) (p < 0.01).

About 16.5% (n = 16) of older adults in COV-g had a risk of frailty associated with UIWL after the disease, while 2.6% (n = 5) of NCOV-g had this risk (OR = 7.35; p < 0.01).

The mean difference in systolic blood pressure (SBP) was also greater among older adults who had the disease (1.33 + 4.75 mmHg vs. 0.13 + 5.28 mmHg, respectively; p = 0.02; Cohen’s d = 0.94) (Table 3).

In covariance analysis, it was observed that after six months of positive diagnosis for COVID the disease was associated with greater weight reduction (F(11.83) p < 0.01; ω^2^ = 0.02), reduced BMI (F(13.55); p < 0.01; ω^2^ = 0.02), increased SBP (F(4.34); p = 0.04; ω^2^ = 0.16), and increased diastolic blood pressure (DBP) (F(4.83); p = 0.03; ω^2^ = 0.02) (Table 3).

## DISCUSSION

This research presented an analysis of the health impacts of COVID-19 on institutionalized older adults six months after a positive diagnosis of the disease. It noted that the effects of SARS-CoV-2 infection go beyond the immediate complications associated with the disease. It is worth noting that accurate data from residents of NHs are scarce, and several countries, including Brazil, have worked with estimates, and this is an important differential of this study, which was conducted with primary data^(19)^.

The sociodemographic and lifestyle profile of older adults residents in NHs found by other researchers^(20,21)^ is similar to that observed in this research, where almost half of older adults were 80 years old or older, with most of them being female and with low education. Notably, in Brazil, this profile is expected for older adult residents in these institutions, since these people tend to arrive at NHs older, with some physical and/or cognitive limitations^(20,21)^.

In this study, a small percentage of older adults used tobacco or alcohol, a favorable condition for minimizing the risk of COVID-19 complications in this population, since evidence shows that the SARS-CoV-2 infection harm is associated with tobacco and alcohol consumption^(22)^. Despite the low frequency of people who reported using these substances, a high proportion reported not practicing regular physical activity, increasing the exposure of this population to risk factors for complications of the disease. According to a study by Sallis et al., physical inactivity and increasing the risk of clinical complications of SARS-CoV-2 infection may also favor functional disabilities earlier^(22)^.

Although a high frequency of dependent older adults residing in these institutions was observed, this percentage was even higher after six months of COVID-19 diagnosis. This finding corroborates the data presented by Greco *et al*., and suggests that COVID-19 may accelerate the impairment of physical performance and frailty in institutionalized older adults by up to 20%^(23)^. These researchers observed that older adult survivors of COVID-19 tended to have greater functional dependence with lower grip strength and lower gait speed, making them more dependent after the disease.

UIWL was more significant in older adults diagnosed with COVID-19. Other authors also observed that COVID-19 negatively impacts the body weight and nutritional status of patients diagnosed with the disease, where almost 30% of the assessed patients lost more than 5% of baseline body weight^(24)^, a clinical condition that becomes even more worrisome in older adults, given the close relationship between compromised nutritional status, loss of muscle mass, and frailty.

Among the mechanisms that may explain weight loss in older adults with COVID-19 are systemic inflammation and impaired protein energy intake, associated with symptoms of anosmia, dysgeusia, and anorexia frequently reported by these patients. The inflammation existing in SARS-CoV-2 infection induces the release of cytokines that produce an acute inflammatory state, contributing to reducing muscle mass and strength^(24)^, making older adults susceptible to developing sarcopenia, dependence, and frailty, and therefore may increase the risk of mortality from COVID-19^(25,26)^.

In group x time interaction analysis, it was identified that after six months of COVID-19 diagnosis, the chance of older adults being classified as functionally dependent increased, and this result may be a consequence of a prolonged time of bed confinement after diagnosis, in addition to the possibility of neural, musculoskeletal or pulmonary involvement^(27)^. It is worth noting that symptoms during and after COVID-19 include some disabling conditions such as myalgia, severe hypoxemia, and weight loss, which can be reversed with practical care and rehabilitation actions^(28)^.

Besides favoring dependence and weight loss, increasing the risk of frailty in institutionalized older adults, SARS-CoV-2 infection also compromised some clinical parameters assessed, such as increased SBP and DBP. It is essential to highlight that, in these institutions, the frequency of hypertensive older adults is high, and BP is difficult to control, being easily altered or dysregulated after aggressive events^(29,30)^.

The central axis of hypertension control is associated with the renin-angiotensin-aldosterone system (RAAS) from the signaling of angiotensin II-converting enzyme (ACE2) receptors, the primary ligand of the S protein of SARS-CoV-2. Consequently, cellular structures that express ACE2 can absorb and potentiate SARS-CoV-2, causing a hyperinflammatory state and activation of immune cells, compromising BP control^(29,30)^.

Considering the above, we highlighted the importance of a broad health assessment of older adults affected by COVID-19, especially those residing in institutions. Therefore, it is recommended to maintain a systematic and constant assessment of their health conditions and functional capabilities after the illness, with the aim of restoring the health of these people as quickly as possible.

Given the above, studies of the nature of this research are essential to clarify that the impacts of COVID-19 go beyond the clinical control of the disease in the acute phase but can extend over a long period. These results offer elements for planning older adults’ health care after SARS-CoV-2 infection to minimize the functional impairment of this population and all the consequences resulting from this clinical condition. This study has two significant limitations, one of which is the use of estimated anthropometric measurements (weight and height); however, given the physical limitations of many older adults to use the assessment equipment, sensitive and validated predictive equations were used for this public, minimizing sample losses. The other limitation refers to the unavailability of clinical and anthropometric data of older adults from two institutions that did not agree to continue the research; however, the final sample size of this study remained representative of the population of institutionalized older adults in Maceió-AL.

## CONCLUSION

SARS-CoV-2 infection in this population contributed to impaired nutritional status, functional capacity and increased BP after six months of infection, intensifying the risk of frailty in this population and making them even more vulnerable to opportunistic infections.

Because of the above, it is clear that the effects of COVID-19 are not limited to the acute phase of the disease but can extend over a long period, especially in older adults living in NHs, which can progress to death, compromising their functional capacity or even triggering decompensation of previously existing and previously controlled chronic pathological processes.
